# Supplementary material for: Exploring the use of social media and online methods to engage persons with lived experience and healthcare professionals in creating research agendas: Lessons from a pediatric cancer research priority-setting partnership
Source: PLOS Digit Health. 2024 Jan 8;3(1):e0000181. doi: 10.1371/journal.pdig.0000181 (PMC10773937; doi:10.1371/journal.pdig.0000181)
Supplement: Attachment — Submitted filename: Table - Copy.docx [file pdig.0000181.s001.docx]

**Table:** Details of manuscript revisions

| **Journal Requirements** | **Comment/Modification** |
| --- | --- |
| 1. Please amend your detailed Financial Disclosure statement. This is published with the article. It must therefore be completed in full sentences and contain the exact wording you wish to be published.  a. State what role the funders took in the study. If the funders had no role in your study, please state: “The funders had no role in study design, data collection and analysis, decision to publish, or preparation of the manuscript.” b. If any authors received a salary from any of your funders, please state which authors and which funders.  If you did not receive any funding for this study, please simply state: “The authors received no specific funding for this work.”  2. Please provide separate figure files in .tif or .eps format only and remove any figures embedded in your manuscript file. Please also ensure that all files are under our size limit of 10MB.  For more information about figure files please see our guidelines:  https://journals.plos.org/digitalhealth/s/figures  https://journals.plos.org/digitalhealth/s/figures#loc-file-requirements  3. Fig 5: please (a) provide a direct link to the base layer of the map (i.e., the country or region border shape) and ensure this is also included in the figure legend; and (b) provide a link to the terms of use / license information for the base layer image or shapefile. We cannot publish proprietary or copyrighted maps (e.g. Google Maps, Mapquest) and the terms of use for your map base layer must be compatible with our CC-BY 4.0 license.   Note: if you created the map in a software program like R or ArcGIS, please locate and indicate the source of the basemap shapefile onto which data has been plotted.  If your map was obtained from a copyrighted source please amend the figure so that the base map used is from an openly available source. Alternatively, please provide explicit written permission from the copyright holder granting you the right to publish the material under our CC-BY 4.0 license.  Please note that the following CC BY licenses are compatible with PLOS license: CC BY 4.0, CC BY 2.0 and CC BY 3.0, meanwhile such licenses as CC BY-ND 3.0 and others are not compatible due to additional restrictions.   If you are unsure whether you can use a map or not, please do reach out and we will be able to help you. The following websites are good examples of where you can source open access or public domain maps:  * U.S. Geological Survey (USGS) - All maps are in the public domain. (http://www.usgs.gov)  * PlaniGlobe - All maps are published under a Creative Commons license so please cite “PlaniGlobe, http://www.planiglobe.com, CC BY 2.0” in the image credit after the caption. (http://www.planiglobe.com/?lang=enl)  * Natural Earth - All maps are public domain. (<http://www.naturalearthdata.com/about/terms-of-use/>) | Thank you for these important notes.  1. We have created a Funding Disclosure section below our Declarations section and included the following text:  **FUNDING DISCLOSURE**  The study was supported by the CIHR Catalyst Grant in Patient-Oriented Research funding reference #PAO-169422. The funders had no role in study design, data collection and analysis, decision to publish, or preparation of the manuscript.”  2. All figures submitted in .tif and as separate files.  3. The base layer of our map is generated by OpenSteetMap, which is a free wiki world map (free open geographic map) |
| Reviewer #1 |  |
| I am not an expert in this field of social media campaigns for priority setting. However, the paper was interesting to read and provided very detailed descriptions about this PSP. I found the discussion section about the lessons learned and a reflection on the limitations most interesting. Methods are informative for readers who want to do something similar. | Thank you kindly for this remark and your time and effort spent considering this study. |
| The results section is not very relevant in my opinion, because the results are merely descriptive. Without context, the number of hits or views is not that informative. I think there are some elements to add to the results.   For example, what I missed in the results is information about the effort that was put into this campaign (in terms of hours and money for the paid advertisements), so that the claim of “efficiency” compared to other recruitment strategies can be judged better. I am convinced by the authors that their strategy to reach people had an effect and is certainly different from traditional approaches. But I cannot judge the “effectiveness” of the response, based on the data. Would it be interesting, for example, to place the results in a framework (possible an existing one) to summarize the working components of the strategy and its effectiveness? | Thank you for this recommendation. We agree adding context here is very important. We have added the cost spent on advertisements to the Results section, as well as the research staff hours devoted to the project.  Furthermore, thank you for your insightful suggestion to organize the Results section using a framework established for social media research recruitment. We followed a social media recruitment framework developed by Lang et al. (https://doi.org/10.1111/1747-0080.12810), which was based on literature and the author’s prior experiences of conducting digital pediatric research. The Results section is now organized based on the phases of the following framework: (i) planning for social media use as an engagement strategy, (ii) identifying and understanding the different target audiences and developing a strategy accordingly, (iii) developing and designing campaign content, (iv) implementing, monitoring and iteratively refining the campaign strategies, and (v) evaluating the campaign success. |
| It is written in line 87 “methods to assess social media campaign effectiveness“ as outcome of your scoping review. What are these methods and which one has been applied to this study? How is effectiveness and efficiency defined? Especially since for this study not only the number of participants was relevant, but also the diversity of the group. | Thank you for this important comment. We agree that it is difficult to assess campaign effectiveness given the design of this project and the methods utilized. To clarify this point for readers we have changed the terms “effectiveness” and “efficiency” to “engagement”. We believe this term better reflects meaning in this paper. |
| Table 4: column Applicable platforms does not add a lot to your list of recommendations, or at least I do not understand what it intends to show. As you already point out, popular platforms come and go and also their features might change over time. Linking a specific recommendation to a specific platform might be outdated in the near future. Should you, however, decide to keep the specific platforms, I would suggest adding a small column for each platform and putting a symbol/x in the cells. Then it will be easier to see which recommendations apply to which platform. (currently, they are not in the same order in all cells) | We agree with this important comment and note that even since we submitted this paper, the landscape of possible platforms has shifted. With this in mind, we have removed the “platform” column and made the recommendation list agonistic to platform-type. |
| For readability, I hope in the final paper all figures can be added as a supplemental data. | Thank you for this recommendation. We are very happy to move figures to the supplemental data section as necessary. |
| Minor: in abstract the abbreviation PSP is not introduced | We have changed all versions of the abstract to define PSP. |
| Last. I think an extra (positive) effect of this broad campaign is that many more people know about the topic, even though they do not participate in the prioritization effort. At least they know that this policy is using the ’voice of the people’, which might give them confidence that, ultimately, the research money will be well-spent. In that regard, the large number of people that do not respond to the social media posts should not be considered a waste. This aspect of using social media could be stressed as well. | Thank you for this important point and its relevance to patient- and public involvement and engagement in research. We have added a comment in the discussion to highlight this very useful thought. |
| Reviewer #2 |  |
| I really enjoyed going through the paper. It is heartening to learn how the team has strategically leveraged social media to engage with participants. I, however, would request the authors to add more information into the manuscript along with some edits to consider. I have enlisted these below: | Thank you kindly for this remark and your time and effort spent considering this study. |
| 1. Please add a definition for 'knowledge user'. I struggled a bit trying to differentiate it from the word 'stakeholder' and 'social media user'. At places in the manuscript, I felt as if the 'knowledge user' and the 'social media user' words could be/ have been used interchangeably though there were some eligibility criteria set by the authors. Even these eligibility criteria have not been specified conspicuously in the paper. | Thank you for this comment. The term knowledge user has been removed and replaced with persons with lived experience and healthcare professionals to clearly delineate our meaning.  We also removed mention of “social media users” to enhance clarity in the paper. |
| 2. The authors have generated the learnings in the process of 'experimenting' strategies with the social media campaigning. However, it is unclear how these learnings were consolidated. This should come up as a paragraph in the methods section. Was it an iterative consensus building effort by the team among themselves? Were these consolidated learnings validated in anyway with the help of domain experts e.g. social media marketing experts, campaign experts, etc? | Thank you so much for this valuable question. Our social media strategies were consolidated and validated through literature. Specifically, we conducted a scoping review exploring effective social media strategies for recruiting for a research project of our nature (doi: 10.2196/29821) - which we know cite in the present paper. Further, throughout our campaign duration, we followed a framework developed by Lang et al. (https://doi.org/10.1111/1747-0080.12810), which was based on literature and the author’s prior experiences of conducting digital pediatric research. As you have suggested, we have edited the Methods section to reflect this consolidation process more explicitly. |
| 3. As I read through the paper, I could appreciate the things that worked. But, what were the strategies that did not work and had to be phased out in the process. Of course, I could get that engaging with people from Quebec has been a challenge despite targeted efforts but did the team 'phase out' any particular effort? | Thank you for this comment. In the results section, we have added text describing strategies we phased out (which aligns with the revision component of Lang et al’s framework). We further review this piece of the study in the discussion section. |
| 4. Please provide the details of the team that managed the strategy e.g. number of members, their profiles, and if there were any prior experience that the team had in designing/ managing such campaigns. | Thank you. Details on these persons have been added to the methods section. |
| 5. I understand that some of the interventions e.g. placing paid ads were added to the strategy in due course. Could it be possible to include a flow diagram for a timeline so that the reader knows the total duration of the campaign and what was done when along with respective outputs? | This is a very important remark. Such details were presented in Figures 3a and 3b, but were not adequately legible. We have reworked these figures to improve readability and highlight where our campaign manuevers were made. |
| 6. Please add the details of the each 'member category' (lines 72-73), Canadian Pediatric Cancer Community (line 75), full description paper (Line 79; if unavailable, please add a brief summary as a supplementary file), 'other online tools' (line 79-80), social media profiles that allowed 'business/ professional accounts' (line 99-100), 'key childhood cancer organizations (Line 110; how were these identified? Were these only from Canada?), 'social media users' (line 110; how were these identified?) | Thank you - we have clarified the meaning of each of these terms. |
| 7. In line 131, the authors have identified that video posts were 'cost-effective'. How was this adjudged? Was any cost-effectiveness analysis done? What was the cost of the overall campaign and its specific elements? | We agree adding context here is very important. We have added the cost spent on advertisements to the Results section, as well as the research staff hours devoted to the project per campaign phase.  We did not conduct a cost-effectiveness analysis and therefore have changed our language in the paper. |
| 8. Could the sentence in line 178 "social media campaigning efforts.... website" in the Results section be moved to the Methods section instead? | Yes – thank you. This sentence fits much better in the Methods section and i now moved there. |
| Once the authors add the above details, the paper will be very detailed to guide the reader interested in emulation.  Thank you! | Thank you very much. |
